# AI predictive models and advancements in microdissection testicular sperm extraction for non-obstructive azoospermia: a systematic scoping review

**DOI:** 10.1093/hropen/hoae070

**Published:** 2024-11-21

**Authors:** Hossein Jamalirad, Mahdie Jajroudi, Bahareh Khajehpour, Mohammad Ali Sadighi Gilani, Saeid Eslami, Marjan Sabbaghian, Hassan Vakili Arki

**Affiliations:** Department of Medical Informatics, Faculty of Medicine, Mashhad University of Medical Sciences, Mashhad, Iran; Department of Medical Informatics, Faculty of Medicine, Mashhad University of Medical Sciences, Mashhad, Iran; Pharmaceutical Research Center, Mashhad University of Medical Sciences, Mashhad, Iran; Midwifery Department, Faculty of Nursing and Midwifery, Mashhad University of Medical Sciences, Mashhad, Iran; Department of Andrology, Reproductive Biomedicine Research Center, Royan Institute for Reproductive Biomedicine, ACECR, Tehran, Iran; Department of Urology, Shariati Hospital, Tehran University of Medical Sciences, Tehran, Iran; Department of Medical Informatics, Faculty of Medicine, Mashhad University of Medical Sciences, Mashhad, Iran; Pharmaceutical Research Center, Mashhad University of Medical Sciences, Mashhad, Iran; Department of Andrology, Reproductive Biomedicine Research Center, Royan Institute for Reproductive Biomedicine, ACECR, Tehran, Iran; Department of Medical Informatics, Faculty of Medicine, Mashhad University of Medical Sciences, Mashhad, Iran

**Keywords:** male infertility, microdissection testicular sperm extraction, non-obstructive azoospermia, artificial intelligence, successful sperm retrieval

## Abstract

**STUDY QUESTION:**

How accurately can artificial intelligence (AI) models predict sperm retrieval in non-obstructive azoospermia (NOA) patients undergoing micro-testicular sperm extraction (m-TESE) surgery?

**SUMMARY ANSWER:**

AI predictive models hold significant promise in predicting successful sperm retrieval in NOA patients undergoing m-TESE, although limitations regarding variability of study designs, small sample sizes, and a lack of validation studies restrict the overall generalizability of studies in this area.

**WHAT IS KNOWN ALREADY:**

Previous studies have explored various predictors of successful sperm retrieval in m-TESE, including clinical and hormonal factors. However, no consistent predictive model has yet been established.

**STUDY DESIGN, SIZE, DURATION:**

A comprehensive literature search was conducted following PRISMA-ScR guidelines, covering PubMed and Scopus databases from 2013 to 15 May 2024. Relevant English-language studies were identified using Medical Subject Headings (MeSH) terms. We also used PubMed’s ‘similar articles’ and ‘cited by’ features for thorough bibliographic screening to ensure comprehensive coverage of relevant literature.

**PARTICIPANTS/MATERIALS, SETTING, METHODS:**

The review included studies on patients with NOA where AI-based models were used for predicting m-TESE outcomes, by incorporating clinical data, hormonal levels, histopathological evaluations, and genetic parameters. Various machine learning and deep learning techniques, including logistic regression, were employed. The Prediction Model Risk of Bias Assessment Tool (PROBAST) evaluated the bias in the studies, and their quality was assessed using the Transparent Reporting of a Multivariable Prediction Model for Individual Prognosis or Diagnosis (TRIPOD) guidelines, ensuring robust reporting standards and methodological rigor.

**MAIN RESULTS AND THE ROLE OF CHANCE:**

Out of 427 screened articles, 45 met the inclusion criteria, with most using logistic regression and machine learning to predict m-TESE outcomes. AI-based models demonstrated strong potential by integrating clinical, hormonal, and biological factors. However, limitations of the studies included small sample sizes, legal barriers, and challenges in generalizability and validation. While some studies featured larger, multicenter designs, many were constrained by sample size. Most studies had a low risk of bias in participant selection and outcome determination, and two-thirds were rated as low risk for predictor assessment, but the analysis methods varied.

**LIMITATIONS, REASONS FOR CAUTION:**

The limitations of this review include the heterogeneity of the included research, potential publication bias and reliance on only two databases (PubMed and Scopus), which may limit the scope of the findings. Additionally, the absence of a meta-analysis prevents quantitative assessment of the consistency of models. Despite this, the review offers valuable insights into AI predictive models for m-TESE in NOA.

**WIDER IMPLICATIONS OF THE FINDINGS:**

The review highlights the potential of advanced AI techniques in predicting successful sperm retrieval for NOA patients undergoing m-TESE. By integrating clinical, hormonal, histopathological, and genetic factors, AI models can enhance decision-making and improve patient outcomes, reducing the number of unsuccessful procedures. However, to further enhance the precision and reliability of AI predictions in reproductive medicine, future studies should address current limitations by incorporating larger sample sizes and conducting prospective validation trials. This continued research and development is crucial for strengthening the applicability of AI models and ensuring broader clinical adoption.

**STUDY FUNDING/COMPETING INTEREST(S):**

The authors would like to acknowledge Mashhad University of Medical Sciences, Mashhad, Iran, for financial support (Grant ID: 4020802). The authors declare no competing interests.

**REGISTRATION NUMBER:**

N/A.

WHAT DOES THIS MEAN FOR PATIENTS? Non-obstructive azoospermia is a severe form of male infertility where no sperm is present in the semen due to issues in the testes. This condition can be very challenging for couples wishing to have biological children. One of the most effective treatments is microdissection testicular sperm extraction (m-TESE), a surgical procedure that involves carefully searching the testes to find and extract sperm that can be used for *in vitro* fertilization. However, not all m-TESE procedures are successful, which can cause physical, emotional, and financial burdens for patients. Using artificial intelligence (AI) to predict the likelihood of successful sperm retrieval before m-TESE might significantly improve preoperative planning and patient counselling. This is where AI can help. AI can analyze various factors such as clinical data, hormone levels and genetic information to predict the success of sperm retrieval. Our review looked at studies from the past decade that have used AI to predict the outcomes of m-TESE in patients with non-obstructive azoospermia. We found that AI methods, including machine learning and deep learning, show great promise in improving these predictions. By considering a wide range of factors, AI can help doctors and patients make more informed decisions, potentially reducing the risks and improving the chances of successful treatment. The findings from this review highlight the importance of continuing research in this area. Better predictive models can lead to better patient outcomes, lessening the physical and emotional toll of infertility treatments and providing hope to many couples.

## Introduction

### Overview

Infertility is a significant global concern that impacts over 100 million individuals generally. It is believed that male-related factors contribute to as much as half of these instances, and this percentage has been on the rise in recent years (Cherouveim *et al.*, 2023). Many clinical factors are linked to male infertility, and numerous studies have emphasized that lifestyle and environmental influences may affect sperm characteristics and diminish semen quality. These influences comprise tobacco, alcohol, drug use, stress, obesity, and sleep deprivation, as well as environmental factors like air pollutants and heavy metals. Additionally, prolonged periods of sitting (more than 4 h daily) are significantly connected with a higher percentage of immotile sperm (GhoshRoy *et al.*, 2023). Male infertility can stem from various factors affecting the production, transport, or function of sperm (Kresch *et al.*, 2021; GhoshRoy *et al.*, 2023).

### Non-obstructive azoospermia

Azoospermia, a condition characterized by the absence of sperm in ejaculated semen, affects about 1% of men. Azoospermic patients are broadly categorized into obstructive azoospermia (OA) and NOA. This differentiation impacts clinical management and reproductive outcomes as OA preserves spermatogenesis with mechanical obstruction along the reproductive tract (Lantsberg *et al.*, 2022; Deng *et al.*, 2023c). Approximately 40% of azoospermia cases are due to OA caused by a blockage in the vas deferens, while NOA constitutes roughly 60%, resulting from causes such as testicular spermatogenic failure. Successful retrieval of healthy sperm from the testes is essential for managing this condition and enabling patients to have biological offspring. NOA is caused by various factors, including acquired causes like cryptorchidism and exposure to gonadotoxic chemotherapy or pelvic radiation, as well as genetic causes such as Klinefelter’s syndrome and Y chromosome microdeletions (Brant and Schlegel, 2023).

### Assisted reproductive technologies

ARTs consist of various medical procedures aimed at helping couples achieve pregnancy when natural conception is not successful. These may include methods such as intrauterine insemination, *in vitro* fertilization with intracytoplasmic sperm injection (ICSI), and surgical interventions to retrieve sperm for assisted reproduction purposes. However, it is important to note that the success of these interventions may vary depending on the underlying cause of male infertility. Microdissection testicular sperm extraction (m-TESE) has emerged as a significant advancement in the management of males with NOA. This innovative surgical technique allows for the precise identification and extraction of viable sperm from the testes, even in cases where sperm production is severely impaired. m-TESE has revolutionized the options available to couples facing male infertility, particularly those who were previously limited in their treatment choices (Marinaro and Goldstein, 2022). The collaboration between reproductive endocrinologists and reproductive urologists is crucial in optimizing the outcomes of assisted reproductive technologies, especially in cases of male factor infertility. By combining their expertise in hormonal manipulation, surgical interventions, and assisted reproductive procedures, these specialists can provide comprehensive and individualized treatment plans for couples struggling with male infertility.

### m-TESE/sperm retrieval rate (SRR)

The treatment of NOA typically involves surgical interventions to retrieve mature sperm from dysfunctional testes, in combination with ICSI for assisted reproduction. There are three standard surgical procedures: testicular sperm aspiration (TESA), testicular sperm extraction (TESE), and m-TESE. Unlike TESA, which is performed blindly and may miss some spermatogenic regions, m-TESE fully exposes seminiferous tubules in the testicular lobules, allowing for enhanced examination and selective biopsy of thick and full tubules through microscopy. This improves the overall rate of successful sperm retrieval (Deng *et al.*, 2023c).

m-TESE has resulted in an average pooled clinical pregnancy rate of 39% when used for ICSI, with some series reporting rates as high as 72.4%. It is endorsed by both the American Urological Association and the American Society for Reproductive Medicine as the premier approach for sperm retrieval in NOA cases (Brant and Schlegel, 2023).

### Overview of SRR and predictors

m-TESE is widely recognized for its higher sperm retrieval rates compared to TESE, especially in patients with NOA. However, the overall success in achieving pregnancy with testicular sperm retrieved from m-TESE in NOA patients remains inadequately documented (Bachelot *et al.*, 2023). Numerous studies have reported several preoperative predictors, including age, body mass index (BMI), testicular volume, follicle-stimulating hormone (FSH) and luteinizing hormone (LH), anti-Müllerian hormone (AMH), inhibin B (InhB), and testosterone levels, which may help in predicting successful sperm retrieval using m-TESE. However, a tool for successful prediction for any single case has been elusive. Although these biomarkers provide valuable prognostic information, their predictive accuracy in unselected patient populations remains inconsistent. This demonstrates that male infertility is complex, and further research is needed in order to provide more reliable and universally applicable prognostic tools for m-TESE results (Lantsberg *et al.*, 2022).

The causes of NOA are diverse, and the success rates of m-TESE can vary significantly based on the underlying etiology. Patients with Klinefelter’s syndrome have moderate success rates of about 50%, which is higher than those with idiopathic NOA but lower than patients with specific genetic deletions such as Y chromosome azoospermia factor C (AZFc) deletion, where success rates can reach up to 67%. In contrast, deletions in the AZFa and AZFb regions are associated with poor outcomes, often discouraging the use of TESE in these cases. Similarly, patients with a history of cryptorchidism have relatively high success rates of around 62%, while those with idiopathic NOA, the most common form, tend to have the lowest success rates, generally ranging between 30% to 40% (Lantsberg *et al.*, 2022). In addition to genetic and clinical factors, histopathological evaluation of testicular tissue can provide valuable insights into the potential success of m-TESE. Histological patterns such as hypospermatogenesis or maturation arrest (MA), when present as the most advanced spermatogenic pattern, are associated with better outcomes compared to cases with Sertoli cell-only syndrome (SCOS) (Brant and Schlegel, 2023). Nonetheless, histopathological assessment involves invasive procedures that may be uncomfortable or distressing for patients (Deng *et al.*, 2023c). Despite extensive examination, these factors have demonstrated variable effectiveness in predicting successful outcomes, making it challenging to develop predictive models that can better support clinical decision-making and improve patient counseling.

### Artificial intelligence

AI has the potential to revolutionize the field of male infertility by assisting clinicians in developing personalized and precise treatment plans based on individual patient characteristics and predicting treatment outcomes (Guahmich *et al.*, 2023). This can lead to more successful sperm retrievals and improved chances of achieving pregnancy for couples dealing with male infertility.

Machine learning uses computer programs to learn from a training dataset and make predictions within a predefined task. By supplying the computer with datasets and desired outputs, machine learning algorithms are created to predict future outcomes within specific tasks. This integration has been notably successful in medicine due to its ability to handle large amounts of complex medical data with which traditional algorithms struggle (Guahmich *et al.*, 2023). Furthermore, the use of AI and machine learning algorithms in predicting the success of sperm retrieval in men with NOA has shown promising results. Machine learning has emerged as a technology that may potentially expedite and enhance the accuracy of sperm identification (Brant and Schlegel, 2023). Machine learning methods enable the prediction of outcomes by analyzing extensive datasets, benefiting from advancements in computational capabilities. Previous literature has highlighted the significance of mathematical modeling and machine learning strategies. In relation to testicular sperm extraction, various models have been created using standard clinical and biological data obtained during preoperative evaluations. The predominant models include logistic regression or artificial neural network models (Bachelot *et al.*, 2023).

This study intends to investigate the evidence for predicting sperm retrieval in NOA patients undergoing m-TESE surgery using AI by answering “Which learning models are employed to predict the success of m-TESE for NOA patients, and which biomarkers have been highlighted as predictors of success in m-TESE?”.

## Methods

### Study design

This investigation was carried out in accordance with the Preferred Reporting Items for Systematic Reviews and Meta-Analyses Extension for Scoping Reviews (PRISMA-ScR) guidelines. This study was conducted to evaluate the predictive variables and machine learning models in determining the success of sperm retrieval in men with NOA undergoing an m-TESE procedure.

### Search strategy

The research conducted involved a systematic search in PubMed and Scopus to gather all pertinent papers published from the last 11 years up to 15 May 2024. Various combinations of terms were utilized with language restrictions set to English, such as: ‘non-obstructive azoospermia’ OR ‘NOA’ AND ‘AI’ OR ‘machine learning’ OR ‘deep learning’ OR ‘logistic regression’ OR ‘predict*’ AND ‘sperm retrieval*’ OR ‘testicular sperm extraction*’ OR ‘micro-TESE’ OR ‘sperm extraction’ OR ‘testicular sperm extraction’ (Supplementary Table S1). The obtained records were imported into ZOTERO 6.0.30 (Corporation for Digital Scholarship, Fairfax, VA, USA), and duplicate entries were eliminated. Additionally, the references cited by these studies and those citing them were reviewed for supplementary relevant publications.

### Study selection

The inclusion criteria encompassed studies involving individuals diagnosed with NOA and undergoing m-TESE. These studies could be non-randomized controlled trials, case-control studies, cohort studies, cross-sectional or analytical studies. Furthermore, the studies had to be original human research that involved factors such as clinical variables, histopathological parameters, genetic predictors, and hormone levels.

Based on the exclusion criteria developed for this review, several studies were excluded from the analysis. Studies that focused on populations with other types of male infertility or emphasized clinical outcomes such as ICSI, pregnancy, and live birth rather than successful sperm extraction by microdissection surgery were excluded. Additionally, studies that were based on statistical analysis instead of AI and machine learning methods for prediction were not considered.

Another crucial aspect of the exclusion criteria was the age of the study population. Therefore, studies that involved patients under 18 years old were excluded. In addition, studies published in venues that were not peer-reviewed scientific sources, such as books, book chapters, proceedings, editorials, PhD dissertations, MSc theses, commentaries, and others, were not considered in this review.

Two authors independently screened all potential literature by title and abstract. Disagreements were solved by discussion; if that failed, another author was called on to adjudicate the final judgment. The same authors performed the full-text screening to determine the final selection.

### Data extraction and synthesis

The data extraction into an Excel spreadsheet was carried out and verified by the authors. Any inconsistencies were addressed through discussion and review of the original data. The extracted information included the first author, year of publication, title, single/multicenter(s), sample size, total SRR, machine learning methods, outcome summary, and conclusion drawn from the studies. Additionally noted were any potential research gaps identified in the literature as well as variables of interest such as clinical, hormonal, histopathological factors, and genetic/RNA-related elements along with their reported significance.

### Risk of bias

To ensure the methodological soundness and applicability of the included studies, a thorough evaluation of bias was conducted.

The risk of bias and applicability of each AI model within the included studies were rigorously assessed using the Prediction Model Risk of Bias Assessment Tool (PROBAST).

The Transparent Reporting of a Multivariable Prediction Model for Individual Prognosis or Diagnosis (TRIPOD) statement is a comprehensive set of guidelines aimed at ensuring clear, transparent, and thorough reporting in studies that develop, validate or update prediction models.

PROBAST and TRIPOD, which were applied independently by two reviewers with any discrepancies resolved through discussion, are designed to evaluate the methodological integrity and relevance of multivariable prediction models in clinical research.

## Results

### Search results and characteristics of the included studies

**Figure 1.**
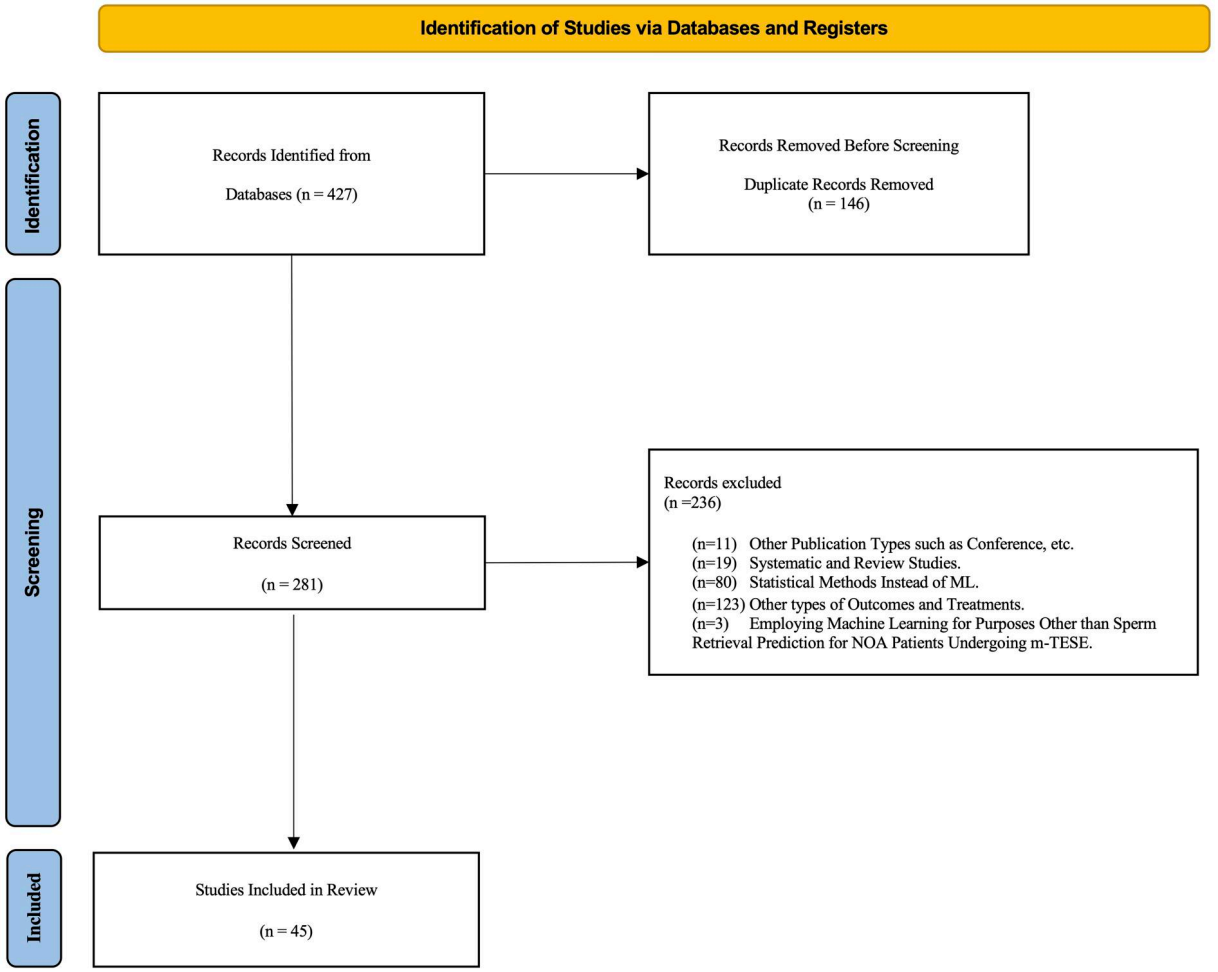
**Flowchart of the study selection process according to the PRISMA-ScR Guidelines.** This flowchart outlines the process of identifying, screening, and selecting studies for inclusion in the systematic scoping review. It details the number of records identified through database searches, records screened, studies assessed for eligibility, and those included in the final review. The flowchart also highlights the number of studies excluded at each stage, with reasons provided for exclusions during the full-text review. PRISMA-ScR, preferred reporting items for systematic reviews and meta-analyses extension for scoping reviews; ML, machine learning; m-TESE, microdissection testicular sperm extraction; NOA, non-obstructive azoospermia.

A total of 45 articles (Fig. 1) were selected from the search on PubMed/Medline and Scopus for utilizing AI techniques in m-TESE for NOA patients. Recent studies have indicated a notable rise in application of machine learning for predicting successful sperm retrieval for m-TESE treatment of NOA. Our analysis, considering relevant inclusion criteria, demonstrates that the most prolific nation publishing research in this area has been China, followed by Italy (Supplementary Fig. S1).

### Patient characteristics

Out of 45 included articles, two specifically focused on the use of deep learning. Data extraction and synthesis were performed on a total of 38 articles, which together covered information from 11 636 patients with NOA who had undergone m-TESE.

### AI and predictive models for m-TESE outcomes in patients with NOA

All the 45 selected articles used at least one AI technique, demonstrating a strong trend towards incorporating advanced technology in the prediction of sperm retrieval effectiveness for patients with NOA undergoing m-TESE. Of 45 articles, 40 focused on primary m-TESE, while five studies addressed salvage m-TESE empowered by AI (Tables 1 and 2). Furthermore, two articles incorporated deep learning methods, reflecting the increasing use of this advanced technique in predicting sperm identification in testicular biopsy samples. The use of these factors bolsters the depth and comprehensiveness of the predictive models, contributing to a more holistic and accurate assessment of sperm retrieval outcomes in patients with NOA. Also, it is important to consider that among the 45 total included articles, seven articles utilized from artificial neural networks (ANN), principal component analysis (PCA), gradient-boost, decision tree (DT), random forest (RF), support vector machine (SVM), extreme gradient-boosting (XGBoost), or deep neural networks (DNN), while the others used logistic regression.

**Table 1.** Studies which have utilized AI techniques to predict success in patients with non-obstructive azoospermia undergoing microdissection testicular sperm extraction.

Author(s) and year	Country	No. of centers	Sample size	Success rate	Area under the curve (AUC)_best model_	Machine learning method	Calibration
Lv *et al.*, 2024	China	Single center	190	SSR: 84/190 SRF: 96/190	AUC_LR_ = 0.997	Logistic regression	Absent

Tian *et al.*, 2023	China	Multicenter	Training: 1292 Ex. validation: 530	SSR: 492/1292 SRF: 800/1292 SSR: 257/530 SRF: 273/530	AUC_RF_ = 0.75	Logistic regression; LASSO, RF, and XG-boost	Absent

Kobayashi *et al.*, 2023	Japan	Single center	430	SSR: 151/430 SRF: 279/430	AUC_ANN_ = 0.72	A gradient-boosting tree and ANN	Absent

Bachelot *et al.*, 2023	France	Single center	175	SSR: 104/175 SRF: 71/175	AUC_RF_ = 0.9	Logistic regression, decision tree, RF, support vector machine, ANN, gradient boosting, extreme gradient boosting, and deep neural network	Yes

Deng *et al*., 2023a	China	Single center	168	SSR: 51/168 SRF: 117/168	AUC_LR_ = 0.76	Logistic regression	Absent

Shi *et al.*, 2022	China	Single center	114	SSR: 47/114 SRF: 67/114	AUC_LR_ = 0.83	Logistic regression	Yes

Zhang *et al.*, 2023 a	Canada	Multicenter	122	Not mentioned	1–0.87	Data mining	Absent

Pozzi *et al.*, 2023	Italy	Multicenter	117	SSR: 57/117 SRF: 60/117	AUC_LR_ = 0.703	Logistic regression	Absent

Zhang *et al.*, 2023b	China	Single center	1030	SSR: 238/1030 SRF: 792/1030	AUC_LR_ = 0.628	Logistic regression	Absent

Kaltsas *et al.*, 2023	Greece	Single center	50	SSR: 22/50 SRF: 28/50	AUC_LR_ = 0.216	Logistic regression	Absent

Willems *et al.*, 2023	Brussels	Single center	38	SSR: 8/38 SRF: 30/38	Not mentioned	PCA	Absent

Cao *et al.*, 2023	China	Single center	96	SSR: 64/96 SRF: 32/96	AUC_LR_ = 0.81	Logistic regression, LASSO	Absent

Deng *et al*., 2023b	China	Single center	181 m-TESE: 116	SSRm: 74/116 SRFm: 42/116	Not mentioned	Logistic regression	Absent

Zheng *et al.*, 2024	China	Single center	Retrospective Analysis (RA): 261 Prospective Analysis (PA): 48	SSR RA: 191/261 SRF RA: 70/261 SSR PA: 11/48 SRF PA: 36/48	0.81	Logistic regression	Absent

Rachman *et al.*, 2023	Indonesia	Single center	517 OA: 164 NOA: 353	SSR: 83/353 SRF: 271/353	Not mentioned	Logistic regression	Absent

Kim and Koo, 2023	Korea	Single center	111	SSR: 68/111 SRF: 43/111	Not mentioned	Logistic regression	Absent

Zhang *et al.*, 2022a	China	Single center	116	SSR: 48/116 SRF: 68/116	AUC_LR_ = 0.927	Logistic regression	Absent

Falcone *et al.*, 2022	Italy	Single center	80	SSR: 22/80 SRF: 68/80	Not mentioned	Logistic regression	Absent

Chen *et al.*, 2022	China	Single center	162	SSR: 78/162 SRF: 84/162	AUC_LR_ = 0.907	Logistic regression	Absent

Aljubran *et al.*, 2022	Saudi Arabia	Single center	108	SSR: 51/108 SRF: 57/108	Not mentioned	Logistic regression	Absent

Lantsberg *et al.*, 2022	Australia	Single center	85	SSR: 52/85 SRF: 33/85	Not mentioned	Logistic regression	Absent

Zhang *et al.*, 2022b	China	Single center	42 30 patient NOA	SSR: 12/30 SRF: 18/30	AUC_LR_ = 0.954	Logistic regression, PCA	Absent

Ji *et al.*, 2021	China	Single center	52	SSR: 20/52 SRF: 32/52	AUC_LR_ = 0.958	Logistic regression, LASSO	Absent

Zhou *et al.*, 2021	China	Single center	62	Not mentioned	AUC_LR_ = 0.786	Logistic regression	Absent

Lee *et al.*, 2022	Canada	Single center	35 761 image patches	Not mentioned	Not mentioned	CNN based on the U-Net	Absent

Wu *et al.*, 2021	USA	Single center	30	Not mentioned	Not mentioned	MobileNetV2, SSD	Absent

Xie *et al.*, 2020	China	Multicenter	96	SSR: 64/96 SRF: 32/96	AUC_LR_ = 0.986	Logistic regression	Absent

Chen *et al.*, 2021	China	Single center	45	SSR: 24/45 SRF: 21/45	AUC_LR_ = 0.82	Logistic regression	Absent


Pavan-Jukic *et al.*, 2020	Slovenia and Croatia	Single center	62	SSR: 31/62 SRF: 31/62	Not mentioned	Logistic regression	Absent

Maglia *et al.*, 2018	Italy	Single center	145 (m-TESE: 49)	SSR: 24/49 SRF: 25/49	Not mentioned	Logistic regression	Absent

Amer *et al.*, 2019	Egypt	Single center	1395	SSR: 450/1395 SRF: 945/1395	AUC_LR_ = 0.682	Logistic regression	Absent

Caroppo *et al.*, 2019	Italy	Single center	143	SSR: 79/143 SRF: 64/143	AUC_LR_ = 0.93	Logistic regression	Absent

Salehi *et al.*, 2017	Iran	Single center	170	SSR**:** 83/170 SRF: 87/170	Not mentioned	Logistic regression	Absent

Klami *et al.*, 2018	Finland	Not mentioned	100	SSR**:** 42/100 SRF: 58/100	Not mentioned	Logistic regression	Absent

Althakafi *et al.*, 2017	Saudi Arabia	Multicenter	421	SRR: 166/421 SRF: 255/421	Not mentioned	Logistic regression	Absent

Cissen *et al.*, 2016	Netherland	Multicenter	1371	SSR: 599/1371 SRF: 772/1371	AUC_LR_ = 0.65	Logistic regression	Absent

Franco *et al.*, 2016	Italy	Single center	64	SSR: 18/64 SRF: 46/64	Not mentioned	Logistic regression	Absent

Enatsu *et al.*, 2016	Japan	Single center	329	SSR: 97/329 SRF: 232/329	Not mentioned	Logistic regression	Absent

Modarresi *et al.*, 2015	Iran	Single center	148	SSR: 35/148 SRF: 113/148	AUC_LR_ = 0.68	Logistic regression	Absent

Ramasamy *et al.*, 2013	USA	Single center	1026	Not mentioned	AUC_ANN_ = 0.59	ANN Logistic regression	Absent

AI, artificial intelligence; m-TESE, microdissection testicular sperm extraction; NOA, non-obstructive azoospermia; OA, obstructive azoospermia; ANN, artificial neural network; AUC, area under the curve; CNN, convolutional neural network; LASSO, least absolute shrinkage and selection operator; PCA, principal component analysis; RF, random forest; RNN, recurrent neural network; SRF, sperm retrieval failure; SRR, sperm retrieval rate; SSR, successful sperm retrieval; SSD, single-shot detector.

**Table 2.** Studies which have employed AI techniques to predict success in patients with non-obstructive azoospermia undergoing salvage microdissection testicular sperm extraction.

Author(s) and year	Country	No. of centers	Sample size	Success rate	Best AUC	Machine learning method	Calibration
Boeri *et al.*, 2022	Italy	Single center	61	SSR: 30/61 (SRF):31/61	Not mentioned	Logistic regression	Absent

Ghalayini *et al.*, 2022	Jordan	Single center	134 (a total of 88, 61 and 40 men underwent two, three and four sperm retrievals, respectively)	SSR2: 65/88 SRF2: 23/88 SSR3: 53/61 SRF3: 8/61 SSR4: 37/40 SRF4: 3/40	Not mentioned	Logistic regression	Absent

Caroppo *et al.*, 2021	Italy	Single center	79	SSR: 30/79 SRF: 49/79	AUC_LR_ = 0.8104	Logistic regression	Absent

Yücel *et al.*, 2018	Turkey	Single center	49	SSR: 21/49 SRF: 28/49	Not mentioned	Logistic regression	Absent

Xu *et al.*, 2017	China	Single center	52	SSR: 20/52 SRF: 32/52	Not mentioned	Logistic regression	Absent

AI, artificial intelligence; m-TESE, microdissection testicular sperm extraction; AUC, area under the curve; SRF, sperm retrieval failure; SRR, sperm retrieval rate; SSR, successful sperm retrieval.

#### Machine learning techniques for predicting m-TESE success

**Figure 2.**
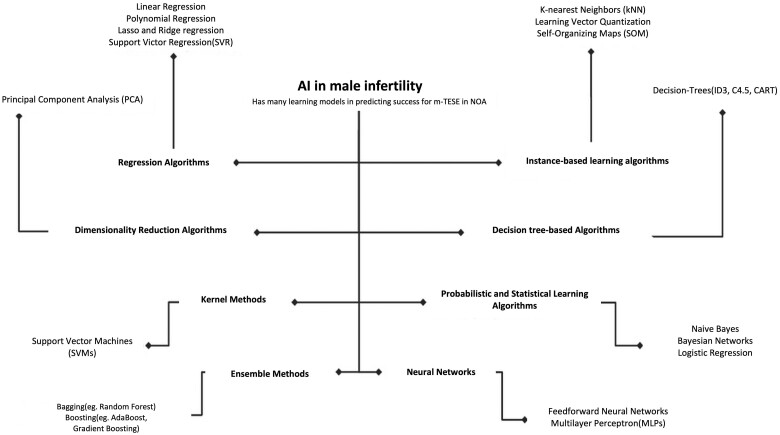
**AI learning models for predicting success in microdissection testicular sperm extraction for non-obstructive azoospermia**. This figure illustrates the application of various AI learning models used to predict the success of m-TESE in men with NOA based on data from male infertility studies. The figure highlights the performance of different models, such as neural networks, support vector machines, and random forests, in terms of accuracy and predictive power. The comparison aims to show which AI model provides the most reliable predictions for successful sperm retrieval in m-TESE. AI, artificial intelligence; m-TESE, microdissection testicular sperm extraction; NOA, non-obstructive azoospermia.

Machine learning models employed to predict sperm retrieval in patients with NOA before m-TESE are illustrated in Fig. 2. Logistic regression, a commonly utilized machine learning algorithm in medicine, enables researchers to assess the impact of multiple variables on an outcome. It is also valuable for comprehending variable relationships and identifying crucial factors in medical research (Boateng and Abaye, 2019).

This approach has been extensively applied to evaluate male infertility and to identify influential factors regarding sperm recovery, as indicated by previous studies using logistic regression (Tables 1 and 2).

Two logistic regression studies on a large patient cohort reported differing outcomes (area under the curve (AUC) = 0.628, AUC = 0.69) compared to similar studies (Cissen *et al.*, 2016; Zhang *et al.*, 2023b). Another study with a sample size of 1395 identified significant associations between sperm recovery and only five parameters out of several including age, infertility duration, FSH level, occupation, residence, lifestyle habits, right testis volume, left testis volume, Sertoli cell only, tubular sclerosis, primary spermatocyte arrest, secondary spermatocyte arrest, spermatid arrest, hypospermatogenesis, hormonal therapy, and type of hormonal therapy (Amer *et al.*, 2019). The complexity and non-linear nature of the data, coupled with the imbalance class in sperm retrieval outcomes, suggest the need for non-linear machine learning approaches like RF and SVM.

Tian *et al.* (2023) aimed to create and assess a predictive model for the clinical outcome of m-TESE in a diverse population with NOA. The study involved 1292 patients as the development cohort and an additional 530 patients for external validation. m-TESE was used for sperm retrieval, and a random forest machine learning method was employed to build a model implemented as a web-based calculator. Results showed that the SRR was 38.1% (492/1292) in the development cohort and 48.5% (257/530) in the validation group. The AUC values indicated good performance; specifically, 0.76 (0.74–0.79) in the development cohort and 0.75 (0.71–0.79) in the external validation, suggesting robustness across cohorts.

Kobayashi *et al.* (2023), a retrospective study involving 430 patients, aimed to develop a model for predicting the likelihood of sperm recovery before m-TESE operation. Results indicated that the neural network model with an AUC of 0.7246 was identified as the most effective in this investigation.

Bachelot *et al.* emphasized the potential of machine learning models to enhance decision support systems in the context of azoospermia by providing more comprehensive information compared to individual variables. Despite exploring various innovative AI models, none were able to conclusively predict TESE results with absolute certainty among the eight different models that were developed, optimized, and assessed. The research involved testing ML algorithms like Bayesian naive classification, logistic regression, *k*-nearest neighbor classifier, support vector machines, random forest, gradient-boosted tree, XGBoost, as well as deep learning models utilizing different neural network architectures. Among all the models, random forest demonstrated superior performance with an AUC of 0.90 (Bachelot *et al.*, 2023).

Ramasamy *et al.* (2013) developed an artificial neural network and nomogram to predict the probability of discovering sperm using m-TESE in men with NOA based on clinical characteristics. The receiver operating characteristics (ROC) area for the neural computational system was 0.641 in the test set and it accurately predicted results for 152 out of 256 patients (59.4%). Ensemble models based on DT, particularly RF, showed the best performance in predicting outcomes. By comparing various machine learning models, the study found that ensemble models based on decision trees, particularly the random forest model, showed the best performance in predicting.

#### Predictors

This research provides a concise overview of the biomarkers and parameters obtained from the study involving four categories encompassing genetics and hormones, as well as histopathological and clinical characteristics. Table 3 illustrates the specific variables that were collected and their corresponding significance.

**Table 3.** Biomarkers of non-obstructive azoospermia patients derived from studies for success prediction in microdissection testicular sperm extraction treatment using AI methods.

Author(s) and year	Variables				
	Clinical	Hormonal	Genetics/micro-RNA/proteins	Histopathology	Importance variable
Lv *et al.*, 2024	Age Testicular volume (TV) Body mass index *(*BMI)	Follicle-stimulating hormone (FSH) Luteinizing hormone (LH) Testosterone (T) Estradiol (E2)	hsa_circ_0058058 hsa_circ_0046188 hsa_circ_0007422 hsa_circ_0003915 hsa_circ_0000965 hsa_circ_0000091 hsa_circ_0004099 hsa_circ_0004463 hsa_circ_0008045 hsa_circ_0001928 hsa_circ_0001179 hsa_circ_0022603 hsa_circ_0065343 hsa_circ_0084789 hsa_circ_0026218 hsa_circ_0007396 hsa_circ_0002211 hsa_circ_0060055 hsa_circ_0000550 hsa_circ_0072088	Not mentioned	hsa_circ_0058058 hsa_circ_0008045 hsa_circ_0084789 hsa_circ_0000550, hsa_circ_0007422 hsa_circ_0004099

Tian *et al.*, 2023	Age TV Etiology (mumps orchitis cryptorchidism) varicocele Surgical history of sperm retrieval	FSH LH T E2 Anti-Müllerian hormone (AMH) Platelet-to-lymphocyte ratio (PLR) Inhibin B (InhB)	Etiology (Y microdeletion AZFc, KS)	Not mentioned	Etiology AMH surgical history of sperm retrieval TV FSH LH Age

Kobayashi *et al.*, 2023	Age height Body weight BMI Left (Lt) varicocele Cryptorchidism Inguinal hernia Torsion of spermatic cord Orchitis Cancer treatment Spinal injury Spina bifida Rt testis size Antisperm antibody (+) Right (Rt) testis, and Lt testis	LH FSH Prolactin (PRL) Total testosterone (tT) E2 Testosterone (T)/E2	G-band (XY 47, XXY 46, XX 46, X 47, XXY46, XY 47, XXY 47, XXY mosaicism) AZFc	Not mentioned	T/E2

Bachelot *et al.*, 2023	Age BMI Smoking Cryptorchidism Infection Trauma Gonadotropic therapy Urogenital surgery Varicocele	LH FSH T InhB PRL	Normal karyotype, Y microdeletion AZFc	Not mentioned	FSH InhB LH PRL Age BMI Smoking Y-chromosome microdeletion Cryptorchidism Varicocele

Deng *et al*., 2023a	Age TV	FSH LH T PRL E2 AMH InhB InhB/AMH	Not mentioned	Not mentioned	InhB/AMH

Shi *et al.*, 2022	Age, BMI TV	LH FSH T	circ_MGLL	Maturation arrest (MA) Sertoli cell-only Syndrome (SCOS) Hypo Spermatogenesis (HS)	circ_MGLL HS

Zhang *et al.*, 2023 a	Not mentioned	Not mentioned	Germ cell-specific proteins	Not mentioned	AKAP4+/ASPX+/Hoechst+ assay

Pozzi *et al.*, 2023	Age Partner age BMI Birthweight Smoking Charlson comorbidity index (CCI) Arterial hypertension History of allergies Mean TV Infertility length Semen volume Bilateral m-TESE Neutrophil–lymphocyte ratio (NLR)	FSH LH tT Sex hormone-binding globulin (SHBG) Albumin Circulating free testosterone (cfT) E2 InhB AMH PRL TSH Vitamin D Insulin	Not mentioned	No germ cells MA Johnsen score (JS) Normal parenchyma	AMH

Zhang *et al.*, 2023b	Age TV BMI Surgical approach Etiology (mumps orchitis cryptorchidism cancer-relative chemotherapy idiopathic varicocele)	AMH FSH LH T E2 InhB PRL	Etiology (Y microdeletion AZFc)	Not mentioned	AMH

Kaltsas *et al.*, 2023	Age TV BMI NLR PLR Monocyte-to-eosinophil ratio (MER) Therapeutic testicular biopsy (TTB)	FSH LH T E2 InhB PRL	Not mentioned	Diagnostic testicular biopsy (DTB)	DTB

Willems *et al.*, 2023	Not mentioned	Not mentioned	microRNA in seminal plasma and urine	Not mentioned	Non-significant

Cao *et al.*, 2023	Age BMI Cryptorchidism-associated Idiopathic Idiopathic testicular atrophy Mumps orchitis Varicocele	FSH LH tT InhB	KS AZFc AZFb Seminal plasma ExLncRNA pairs (SPATA42-LOCI00505685) SPATA42-LOCI01929088(XR_927561.2) CCDC37.DT-LOCI00505685 GABRG3.ASI-LOCI00505685 LOC440934-LOCI00505685 LOC440934-LOCI01929088(XR_001745218.1 LOCI01929088(XR_927561.2)-LINC00343)	Not mentioned	CCDC37.DT-LOCI00505685 LOC440934-LOCI01929088 (XR_001745218.1) Age

Deng *et al*., 2023b	Age TV	FSH LH T	Y microdeletion AZFc	Not mentioned	Non-significant

Zheng *et al.*, 2024	Age BMI iNOA Non-iNOA Orchitis Cryptorchidism	FSH LH tT InhB AMH	Y microdeletion AZFc KS	Not mentioned	AMH

Rachman *et al.*, 2023	Age Marriage duration DM Varicocele undescended testicle Longest testicular axis	FSH LH T	Not mentioned	Not mentioned	Varicocele
Kim and Koo, 2023	Age Height Weight BMI DM Hypertention Volume of right testis Volume of left testis Varicocele Hydrocele	FSH LH Free T PRL FHS/LH T/LH SHBG	Not mentioned	Focal spermatogenesis Tubular hyalinization Hypospermatogenesis MA SCO	T/LH Volume of right testis Volume of left testis

Zhang *et al.*, 2022a	Age Partner age	FSH LH tT	Circulating microRNAs	Not mentioned	hsa-miR-34b-3p, hsa-miR-34c-3p, hsa-miR-3065-3p, and hsa-miR-4446-3p

Falcone *et al.*, 2022	Age Smoking TV (left side Right side)	FSH	Not mentioned	HS SCOS Spermatogenic arrest JS Sperm vials stored	HS SCOS Spermatogenic arrest

Chen *et al.*, 2022	Age BMI Varicocelectomy Cryptorchidism (unilateral bilateral) Location (intra-abdominal inguinal suprascrotal) Age of orchidopexy Dominant TV	FSH LH tT	Not mentioned	Not mentioned	Location Age of orchidopexy Dominant TV Cryptorchidism (unilateral bilateral)

Aljubran *et al.*, 2022	Age TV Sperm count Varicocele Undescended testis	FSH LH T	Not mentioned	JS	Age FSH

Lantsberg *et al.*, 2022	Age Etiology (cryptorchidism childhood diseases testicular cancer idiopathic previous chemotherapy)	FSH LH T	Etiology (chromosomal translocation KS AZFc)	Complete hyalinisation HS MA SCO	KS FSH Childhood diseases
Zhang *et al.*, 2022b	Not mentioned	Not mentioned	Circulating plasma exosomal tRFs	Not mentioned	Plasma circulating exosomal tRF-Gly-GCC-002 and tRF-Glu-CTC-005

Ji *et al.*, 2021	Age BMI Semen volume Sperm retrieval site (unilateral bilateral)	FSH LH T	Circulating RNAs	HS MA SCOS	hsa_circ_0000277, hsa_circ_0060394 and hsa_circ_0007773

Zhou *et al.*, 2021	Age TV	FSH LH T PRL E2	Expression of Beclin-1	JS	Expression of Beclin-1 JS

Xie *et al.*, 2020	Not mentioned	Not mentioned	Long non-coding RNAs	Not mentioned	LOC100505685, SPATA42, CCDC37-DT, GABRG3-AS1, LOC440934, LOC101929088 (XR_927561.2) LOC101929088(XR_001745218.1) LINC00343 and LINC00301

Chen *et al.*, 2021	Not mentioned	Not mentioned	EV-piRNAs in seminal plasma	Not mentioned	pir-60351 pir-61927

Pavan-Jukic *et al.*, 2020	Age BMI Smoking TV Tactical biopsy (right, left) Varicocele Surgery	FSH LH T PRL	Chromosomal profile (46, XY 46, XY, inv 46, XY, reciprocal translocation of 11th and 15th ch 46, XY, H63D polymorphism of HFE 46, XY 46, XXY)	Not mentioned	Smoking
Maglia *et al.*, 2018	Age BMI CCI Smoking Varicocele Cryptorchidism	FSH LH T PRL	Not mentioned	HS MA SCOS	MA SCOS

Amer *et al.*, 2019	Age Infertility duration Occupation Residence Special habit TV (right-left) Hormonal treatment Hormonal type	FSH	Not mentioned	Tubular sclerosis 1ry spermatocyte arrest 2ry spermatocyte arrest Spermatid arrest HS Sertoli cell-only (SCO)	FSH Tubular sclerosis Dry spermatocyte arrest Spermatid arrest HS SCO

Caroppo *et al.*, 2019	Age TV Sperm count per tubule caliber pattern Seminiferous tubule caliber pattern	FSH LH tT	Not mentioned	SCO Focal SCO MA HS Hyalinosis (HL) Intraepithelial neoplasia (IN)	Seminiferous tubule caliber pattern Histology

Salehi *et al.*, 2017	Age TV	FSH LH T PRL	Normal karyotyping	SCOS MA	FSH TV

Klami *et al.*, 2018	Age Testicular size Previous needle biopsy Cytotoxic and Radiation Cryptorchidism	FSH LH T PRL	KS AZFc	SCO SA	Testicular size

Althakafi *et al.*, 2017	Age BMI TV Varicocele Bone marrow transplantation Pretreatment with clomiphene or B-HCG Sperm motility	TSH FSH LH PRL T E2	Chromosomal analysis (XXY47)	SCOS HS Early maturation arrest (EMA) Late maturation arrest (LMA) Active/normal spermatogenesis Normal Spermatogenesis (NS)	Age Histopathology
Cissen *et al.*, 2016	Age BMI Smoking behavior Alcohol consumption Duration of infertility TV Cryptorchidism Orchidopexy Idiopathic/others	FSH LH PRL InhB T	KS Y microdeletion AZFc	Not mentioned	Age LH FSH idiopathic NOA AZFc
Franco *et al.*, 2016	Age	FSH LH InhB	Not mentioned	MA Sclera-hyalinosis	Non-significant

Enatsu *et al.*, 2016	Age TV Etiology (idiopathic varicocele) Experience of surgeon	FSH LH T	Etiology (KS Other karyotype abnormalities)	MA SCO HS	Etiology

Modarresi *et al.*, 2015	Age	FSH LH T	Not mentioned	SCOS	Non-significant

Ramasamy *et al.*, 2013	Age Cryptorchidism Varicocele TV	FSH	KS	Not mentioned	Age, KS Cryptorchidism

AI, artificial intelligence; AMH, anti-Müllerian Hormone; AZF, azoospermia factor; BMI, body mass index; CC, clomiphene citrate; CCI, Charlson comorbidity index; cFT, circulating free testosterone; circ_MGLL, circular RNA monoglyceride lipase; DTB, diagnostic testicular biopsy; E2, estradiol; EMA, early maturation arrest; EVs, extracellular vesicles; FSH, follicle-stimulating hormone; HL, hyalinosis; HS, hypo spermatogenesis; IN, intraepithelial neoplasia; iNOA, idiopathic non-obstructive azoospermia; InhB, Inhibin B; JS, Johnsen score; KS, Klinefelter syndrome; LH, Luteinizing Hormone; LMA, late maturation arrest; MA, maturation arrest; MER, monocyte-to-eosinophil ratio; m-TESE, microdissection testicular sperm extraction, NLR, neutrophil-to-lymphocyte ratio; NS, normal spermatogenesis; NOA, non-obstructive azoospermia; PLR, platelet-to-lymphocyte ratio; PRL, prolactin; SA, spermatogenic arrest; SCOS, Sertoli cell-only syndrome; SHBG, sex hormone-binding globulin; ST, seminiferous tubules; T, testosterone; tRFs, transfer RNA-derived fragments; TSH, thyroid-stimulating hormone; tT, total testosterone; TTB, therapeutic testicular biopsy; TV, testicular volume.

##### Clinical factors

Clinical factors like infertility cause, age, testicular size, BMI, and medical history are evaluated in NOA. Studies have conflicting results on the impact of age on m-TESE success. Some found no link, while others suggest orchidopexy age and testicular volume may affect sperm retrieval rates. Research indicates male age does not significantly impact m-TESE results, with highest success in men over 40 years. Obesity does not affect m-TESE sperm retrieval but lowers pregnancy rates. The correlation of success with testicular volume remains inconsistent. Etiology is a key predictor; idiopathic NOA is associated with a poor prognosis, while patients with conditions like cryptorchidism have higher sperm retrieval rates (Qi *et al.*, 2021).

##### Histopathology

Histopathological evaluation also plays an essential role in predicting m-TESE success in NOA patients. Certain patterns, such as hypospermatogenesis, where spermatogenesis is present but reduced, are associated with higher sperm retrieval rates. Conversely, conditions like Sertoli cell-only syndrome (SCOS) and maturation arrest (MA) are linked to lower success rates. While biopsies can provide predictive information, they are not always recommended due to risks like pain, infection, and hematoma. Recent advancements in machine learning have improved the accuracy of predictions by analyzing complex clinical, hormonal, and histopathological data. Integrating histopathological evaluation with hormonal assessment enhances the ability to predict and manage sperm retrieval outcomes, offering a more comprehensive approach to treating NOA patients undergoing m-TESE (Abdullah and Bondagji, 2011; Weedin *et al.*, 2011; Ghanami Gashti *et al.*, 2021).

##### Hormones

Hormone levels are crucial in predicting the success of m-TESE in patients with NOA. FSH and LH are primary indicators, with elevated FSH levels often signaling impaired spermatogenesis and testicular failure. Testosterone is essential for spermatogenesis and sperm maturation, while high prolactin levels can lead to hypogonadism and spermatogenic failure. Inhibin B, produced by Sertoli cells, inversely regulates FSH and correlates with spermatogenic activity, although its predictive efficacy for sperm retrieval remains debated. AMH, involved in spermatogenic cell differentiation, shows potential as a spermatogenesis marker, but further research is needed to establish its role definitively. Collectively, these hormones provide valuable insights into the likelihood of successful sperm retrieval in NOA patients undergoing m-TESE (Qi *et al.*, 2021; Sengupta *et al.*, 2021; Li *et al.*, 2023).

##### Genetic factors

Genetic factors are pivotal in predicting the success of m-TESE in patients with NOA. Key genetic analyses include tests for sex chromosome anomalies like Klinefelter syndrome (KS), XYY syndrome, and Y chromosome microdeletions. Among these, KS and Y chromosome microdeletions are the most common in infertile patients. The Y chromosome contains AZF regions, crucial for spermatogenesis. Microdeletions in the AZFc region are the most prevalent and offer a higher chance of successful sperm retrieval compared to deletions in AZFa or AZFb regions (Glina and Vieira, 2013; Flannigan *et al.*, 2017; Li *et al.*, 2023; Şen *et al.*, 2023).

Recent research has highlighted the significance of microRNAs (miRNAs) as potential non-invasive biomarkers for predicting m-TESE outcomes. miRNAs are small RNA molecules that regulate gene expression and play roles in germ cell development and spermatogenesis. Abnormal miRNA expression in NOA patients suggests their potential for predicting sperm retrieval success. Moreover, miRNAs in seminal plasma are protected from degradation, making them stable markers. Additionally, long non-coding RNAs (lncRNAs), circular RNAs (circRNAs), and tRNA-derived small RNAs (tsRNAs) in seminal plasma are also being explored as biomarkers. These RNAs contribute to spermatogenesis and offer insights into the spermatogenic status, providing valuable non-invasive predictors for m-TESE success (Li *et al.*, 2023; Şen *et al.*, 2023).

#### Machine learning techniques for salvage m-TESE

Salvage m-TESE is a surgical technique increasingly used to extract viable sperm from the testes of men with NOA after unsuccessful prior attempts at sperm retrieval procedures. This advancement in male infertility offers potential reproductive outcomes for individuals with NOA. Subsequent attempts at salvage m-TESE have shown relatively satisfactory rates of retrieving sperm but are not guaranteed. Machine learning methods successfully identified variables linked to SSR with high precision, offering insights into how specific intra-surgical parameters can predict salvage m-TESE outcomes for NOA patients who have previously faced unsuccessful surgical retrieval attempts. The current research leverages machine learning to gain significant benefits, especially in predicting and projecting elements that strongly correlate with the SRR outcome in individuals with NOA (Table 2).

Boeri *et al.* (2022) developed a study proposing m-TESE as a possible remedy for men who have previously undergone unsuccessful classic TESE (c-TESE). While information on this topic is limited, we sought to assess the outcomes and potential factors that could indicate successful salvage m-TESE in a cohort of individuals with previously unsuccessful c-TESE. The analysis encompassed data from 61 men who underwent m-TESE after failed c-TESE between January 2014 and October 2020 at six tertiary-referral centers in Italy. Various assessments were conducted on all participants before the procedures and comprehensive diagnoses from prior TESE were compiled. Descriptive statistics and logistic regression models were utilized to investigate potential predictors of positive sperm retrieval following salvage m-TESE. The research revealed that hypospermatogenesis was independently linked with favorable results during salvage m-TESE, even after adjusting for age and FSH levels. In conclusion, it can be inferred that salvage m-TESE presents as a safe option for NOA patients with previous negative c-TESE findings and may result in an approximately 50% success rate in achieving a sperm retrieval positive status.

Caroppo *et al.* (2021), by utilizing machine learning methods like predictive models, successfully identified variables linked to SSR with a high level of precision. This approach yielded valuable insights into how specific intra-surgical parameters can predict the result of salvage m-TESE, even for NOA patients who have previously faced unsuccessful surgical retrieval attempts. Additionally, employing machine learning facilitated an elucidation and understanding of variability within the results. The capacity of the model to accurately classify 88.3% of cases and clarify 29.7% of variability highlights its effectiveness in analyzing intricate histopathological findings and recognizing key factors contributing to successful SSR outcomes. Notably, exponential moving average (EMA) emerged as a significant factor within this predictive model, demonstrating how machine learning not only enhances comprehension but also provides practical value by highlighting vital histological categories influencing m-TESE outcomes for NOA patients undergoing salvage procedures following failed conventional approaches.

The research of Yücel *et al.* (2018) was to investigate the practical application of salvage m-TESE in individuals diagnosed with NOA, and to determine the factors that affect the presence of spermatozoa during preoperative salvage m-TESE. The study used multivariate analysis to identify and predict factors linked to successful sperm retrieval during a salvage m-TESE procedure. A model incorporating parameters such as age, BMI, history of varicocele, history of cryptorchidism, duration of infertility, results of genetic analysis, and hormone profiles was developed using multiple logistic regression analysis for comparison between patient groups. The results showed that among predictors influencing successful sperm retrieval in salvaging m-TESE within NOA patients who had previously undergone unsuccessful attempts at an m-TESE operation, only pre-operative FSH levels displayed a significant correlation with success rates in salvage m-TESE procedures.

The aims of the research by Xu *et al.* (2017) were to evaluate the practical application and factors for predicting sperm retrieval through salvage m-TESE in NOA patients who have had unsuccessful c-TESE attempts. A total of 52 NOA males underwent salvage m-TESE, and data on various factors such as age, BMI, presence of Klinefelter’s syndrome, varicocele, or cryptorchidism, mean testicular volume, hormonal profile (total testosterone, FSH, LH, inhibin B levels), testicular histology, and surgical duration were gathered and analyzed. Multivariate logistic regression indicated that total testosterone levels and testicular histology were significant predictors for the likelihood of sperm retrieval during salvage m-TESE. In addition, a predictive model was created using multivariate regression analysis to estimate the probability of sperm retrieval. ROC analysis established a cutoff value at 71% predicted probability with a sensitivity of 78.0% and specificity of 72.4%. These results underscore the benefit of utilizing advanced techniques like multivariate regression models to identify important predictors associated with outcome variables in order to inform clinical decisions regarding potential candidates for salvage m-TESE procedures in NOA patients following failed c-TESE attempts.

Ghalayini *et al.* (2022) showed that repeated m-TESE demonstrates a high success rate in retrieving sperm from patients with NOA, particularly following initial successful procedures. Their findings suggest that when cryopreserved testicular sperm for ICSI yields no viable spermatozoa, scheduling a repeat TESE procedure may be warranted. The use of machine learning provides significant advantages in this study for modeling and predicting factors that are closely related to the outcome variable, such as testicular volume and FSH levels. Specifically, our data indicate a correlation between the success rate of repeated m-TESE and testicular volume as well as FSH concentration. Furthermore, preoperative prognostic data can accurately predict cases where m-TESE is most beneficial, especially in instances involving atrophied testicles or SCOS with elevated FSH concentrations.

#### Deep learning in sperm detection and identification for optimizing m-TESE procedures

The accurate detection of sperm within m-TESE samples post-procedurally remains a critical component of the overall treatment process. There is evidence in some studies that deep learning can identify sperm from testicular biopsies with near-human performance. Deep learning improves sperm identification by enhancing image analysis capabilities, increasing accuracy and efficiency, reducing manual effort, and continuously adapting to new data. These advantages make deep learning a powerful tool in the field of male infertility treatment, particularly for identifying sperm in challenging cases such as NOA. Although these methods are typically used during or post-procedurally to find sperm, it is worth noting that these technologies not only support the precise execution of assisted reproductive techniques like ICSI but also contribute to the broader goal of improving outcomes for patients undergoing m-TESE. Advanced deep learning techniques, as demonstrated in studies by Lee *et al.* (2022) and Wu *et al.* (2021), offer significant potential to enhance the identification of rare sperm in testicular biopsy samples. This information is provided for researchers interested in this aspect, but as it is not directly related to the aim of our study, further exploration of this topic is beyond our current scope.

### Risk of bias in included studies

**Figure 3.**
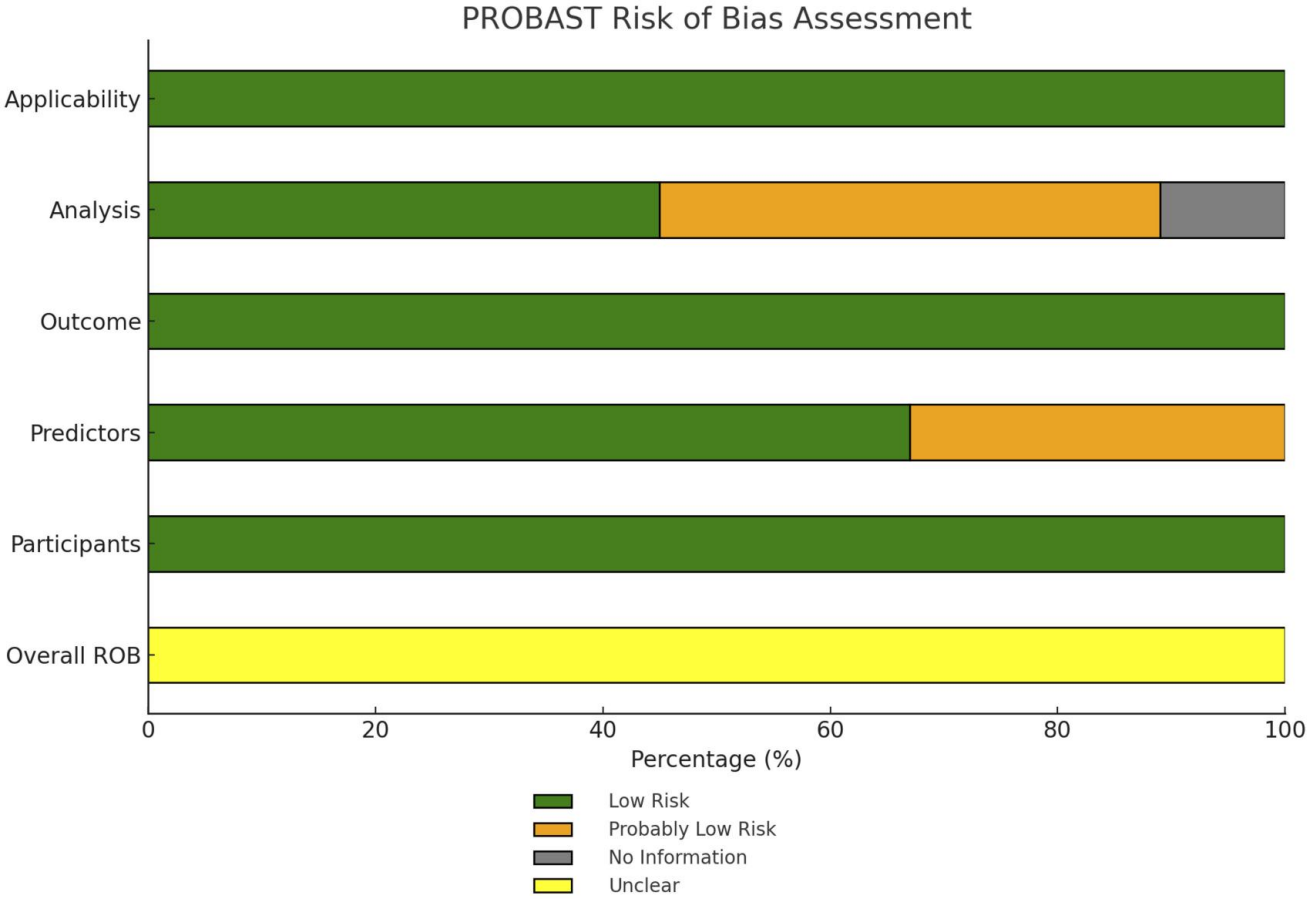
**Overall PROBAST risk of bias assessment for entire included studies predicting sperm retrieval in non-obstructive azoospermia patients undergoing microdissection testicular sperm extraction**. This figure presents the PROBAST assessment of bias risk across key domains in studies focused on predicting sperm retrieval outcomes for NOA patients undergoing m-TESE. The evaluation covers participant selection, predictors, outcomes, analysis, and applicability, with the risk levels indicated by color-coded bars: green (low risk), orange (probably low risk), yellow (unclear), and grey (no information). m-TESE, microdissection testicular sperm extraction; NOA, non-obstructive azoospermia; PROBAST, prediction model study risk of bias assessment tool.

Our review revealed that all included studies demonstrated a low risk of bias and minimal concerns regarding applicability, emphasizing their strong methodological foundation and relevance to clinical practice. Using PROBAST, we evaluated key domains such as participant selection, predictor assessment, outcome determination, and analysis methods. The results showed that 100% of the studies had a low risk of bias in participant selection and outcome determination. For predictor assessment, 33% of studies had a ‘probably low’ risk, while 67% showed a ‘low’ risk of bias. The analysis methods presented more variability, with 44% of studies rated as ‘probably low’, 45% as ‘low’, and 11% categorized under ‘no information’, reflecting varying levels of analytical rigor. Figure 3 and Supplementary Fig. S2 provide a detailed breakdown of these assessments, underscoring the reliability and clinical significance of the reviewed studies. The Cohen’s kappa for interrater agreement was 0.87.

In addition, the transparent reporting of TRIPOD guidelines were used to ensure thorough and transparent reporting across all studies. The TRIPOD checklist, covering 27 essential items from title to discussion, was applied to evaluate the reporting quality. By adhering to these guidelines, the studies ensured clarity, reproducibility, and critical appraisal. Each study’s TRIPOD score is detailed in Supplementary Fig. S3, offering an overview of how well each article met the reporting standards. This comprehensive evaluation of both bias and reporting quality highlights the overall robustness, transparency and reliability of the multivariable prediction model studies reviewed.

## Discussion

Treating patients with NOA often involves a procedure known as m-TESE, which has proven to be highly successful in retrieving sperm, with success rates reaching up to 64% for eligible cases (Glina and Vieira, 2013).

Accurately predicting the success of m-TESE plays a vital role in the effective management of NOA. This research underscores the value of machine learning models in refining preoperative predictions of sperm retrieval, thereby aiding in better patient counseling and surgical decision-making. By incorporating clinical, hormonal, and genetic factors, these models enable a more precise evaluation of which NOA patients are most likely to benefit from m-TESE. Our findings indicate a significant reliance on machine learning techniques across the studies reviewed, highlighting their ability to synthesize complex datasets and deliver predictive insights. This approach has the potential to minimize unnecessary surgeries and enhance treatment outcomes.

Notably, 45 of these studies employed machine learning methods, showcasing a predominant reliance on this type of AI technique in their analyses, emphasizing the multifaceted approach adopted in the prediction models. In predicting successful sperm retrieval prior to m-TESE, studies have reported varied outcomes when employing AI and machine learning algorithms. Logistic regression emerges as the most prevalent algorithm, particularly favored for its simplicity and efficiency in handling big datasets, especially within single-center studies. Consequently, models developed using preoperative clinical, biological, and histopathological data exhibit considerable variability in predictive accuracy. Reported AUC values range widely, from a low of 0.216 (Kaltsas *et al.*, 2023), reflecting significant challenges in smaller, more diverse populations, to a high of 0.997 (Lv *et al.*, 2024), indicative of near-perfect performance in more controlled single-center environments. In more complex multicenter datasets, advanced machine learning techniques, such as random forests, support vector machines, and artificial neural networks, have been employed to enhance model generalizability and predictive performance. While some studies have reported promising results with AUCs reaching up to 0.90, these advanced predictive models still encounter difficulties in maintaining consistent accuracy across diverse patient populations, with AUCs ranging from 0.59 to 0.9. This variability is influenced by factors such as sample size, population diversity, and study design. These findings strongly advocate for rigorous study designs, careful model selection, and comprehensive validation processes to develop AI models that are not only accurate but also broadly applicable for predicting m-TESE outcomes. High AUC scores signify robust model performance but do not guarantee flawless or absolute outcomes due to the intrinsic uncertainty inherent in medical predictions. Information is presented based on the data summarized in Table 1 (Bachelot *et al.*, 2023; Kobayashi *et al.*, 2023; Tian *et al.*, 2023).

Deep learning techniques, particularly convolutional neural networks, have shown potential in automated sperm identification and selection in testicular biopsy samples (Wu *et al.*, 2021; Lee *et al.*, 2022).

The research underlines that the prediction of SRR should consider several combined parameters because no single parameter has been found to be strongly predictive of the success of m-TESE. It has been identified that the most important predictors include testicular histology, genetic markers, hormone levels, and clinical characteristics. As summarized by Table 3, some studies have noted the importance of etiology, age, and testicular volume, while others found those biomarkers to be less predictive. In addition, histopathological patterns also show predictive potential for success in sperm retrieval; hypospermatogenesis shows more chance of sperm retrieval than MA and SCOS. However, the risks associated with diagnostic biopsies limit the spread of use.

The contribution of hormonal factors in predicting SRR is controversial. Whereas some studies have shown that FSH, LH, testosterone, and AMH were correlated with SRR status, others have demonstrated no such significant associations. Furthermore, AMH has been suggested as a potential marker for spermatogenesis in azoospermic men; however, results are diverse. Genetic factors are especially important in predicting the success of m-TESE in NOA patients, including sex chromosome abnormalities such as Klinefelter syndrome and Y chromosome microdeletions in the AZF regions. Genetic studies have promising potential, but they are yet to be incorporated into day-to-day practice due to issues such as complexity, the cost of genetic testing, and the extent of validation in different populations. The emerging advances in non-invasive biomarkers, including microRNAs and other non-coding RNAs, offer some promise for enhanced predictive capabilities but must first pass through clinical testing and standardization procedures before being introduced in practice to improve routine infertility diagnostics and treatment planning. These insights are detailed in Table 3.

Therefore, further research and progress in the field are necessary to enhance patient selection through clinical, hormonal, histopathological, and genetic factors. This includes improving retrieval rates and streamlining the process of sperm identification and analysis. Considering all these variables for predicting outcomes emphasizes the significance of complexity and precision in determining the most suitable treatment approach for patients with NOA. The utilization of advanced AI methods, such as machine learning and deep learning, to predict the effectiveness of sperm retrieval underscores how technology is increasingly influencing clinical decision-making and patient care within male infertility management. It is worth noting that the use of calibration information in predictive modeling within original research articles helps to ensure that the model’s predictions are not only accurate but also interpretable and reliable for practical applications. Our review identifies that only two (Bachelot *et al.*, 2023, Shi *et al.*, 2022) articles have included calibration data in their assessment of predictive model performance. The remaining articles, however, did not discuss the absence of calibration data nor provided any evaluation concerning calibration. This represents a significant gap in the literature, as calibration is crucial for determining how well predicted probabilities align with actual outcomes in clinical settings. Without evaluating calibration, the generalizability of these predictive models is questionable. Consequently, future research should focus on incorporating both discrimination and calibration measures to ensure robust model validation, ultimately improving their utility and reliability in clinical practice. Moreover, the studies acknowledged several limitations, including the need for validation studies, modeling-related constraints, legal barriers in certain countries, calls for prospective randomized control trials, the consideration of additional predictors, and sample size limitations. Addressing these limitations could enhance the accuracy and generalizability of AI-based prediction models in male infertility management.

### Limitations of SRR prediction studies

While this study provides a comprehensive overview of AI predictive models in m-TESE for NOA, it is important to acknowledge certain limitations. The heterogeneity of the included studies, varying in population characteristics, methodologies, and machine learning models, may affect the comparability and generalizability of the findings. Additionally, as the review focused solely on two databases, PubMed and Scopus, this could limit the scope of included studies, potentially overlooking relevant research from other databases. Moreover, the potential for publication bias remains, as studies with inconclusive or negative results may be underrepresented. Finally, the absence of a meta-analysis in this scoping review means that while a broad overview is provided, it lacks the statistical synthesis to quantitatively assess the consistency of the predictive models across studies.

Despite advancements in sperm recovery prediction models and the identification of influential factors using AI to assist doctors in determining whether to initiate or continue m-TESE treatment, this remains a complex issue with limitations within existing studies. The articles covered in this review outline six main categories of limitations and suggestions for further research (Supplementary Table S2).

First, there is a recommendation for validation studies to explore the generalizability and assess the effectiveness of the proposed models for predicting successful sperm recovery.

Second, modeling-related constraints include conducting retrospective studies, challenges associated with data collection, the presence of confounding factors and biases (such as patient selection), along with clinical and laboratory techniques impacting sperm recovery rates.

Third, limitations due to legal or regulatory barriers exist in certain countries, regarding investigation into infertility among men. It is also suggested that sample preparation processes should be simplified and standardized.

Fourth, there are calls for prospective randomized controlled studies intended to validate the current findings; these trials must be carefully designed to adhere to ethical principles. Rather than denying m-TESE treatment based on AI predictions, such RCTs could focus on comparing different approaches where AI-based predictions are used to guide supplementary diagnostic testing or preparatory interventions, aiming to improve patient outcomes without compromising access to care.

Fifth, the inclusion of emerging and less commonly utilized predictors such as microRNAs, Y chromosome microdeletions, and specific histopathological patterns, in conjunction with traditional markers like hormonal levels and genetic factors, can offer better insights into identifying individuals who would benefit most from m-TESE surgery.

Finally, there have been sample size limitation issues, such as small numbers or a lack of samples collected from multiple centers. By addressing these limitations and filling research gaps, future AI-based approaches toward male infertility could significantly enhance diagnostic accuracy, personalized treatment strategies and overall patient outcomes.

## Conclusion

In conclusion, the best use of advanced AI techniques holds a lot of promise in improving the prediction of sperm retrieval success in individuals with NOA. New algorithms based on random forests and support vector machines have great potential for the identification of predictive biomarkers, selection of treatment at an optimal level, and assessment of therapeutic outcomes. Some critical remaining challenges include the need for proper validation studies, overcoming some intrinsic limitations of actual modeling approaches, legal or regulatory barriers in some jurisdictions, and conducting prospective randomized trials. In addition, emerging predictors and the challenge of small sample sizes need to be considered. Overcoming these challenges represents an enormous opportunity for future research to advance diagnostic accuracy, personalize strategies in treating cases, and ultimately improve patient outcomes in the management of male infertility.

## Supplementary Material

hoae070_Supplementary_Data

## Data Availability

The data presented in this article were obtained solely from the original published sources. No additional data were generated or analyzed to substantiate the content of this article. Correspondence and requests for materials should be addressed to M.S. or H.V.A.
