# Supplementary material for: AI predictive models and advancements in microdissection testicular sperm extraction for non-obstructive azoospermia: a systematic scoping review
Source: Hum Reprod Open. 2024 Nov 21;2025(1):hoae070. doi: 10.1093/hropen/hoae070 (PMC11700607; doi:10.1093/hropen/hoae070)
Supplement: hoae070_Supplementary_Data [file hoae070_supplementary_data.zip › HRO-24-0251-R2-SuppFigures1-3.docx]

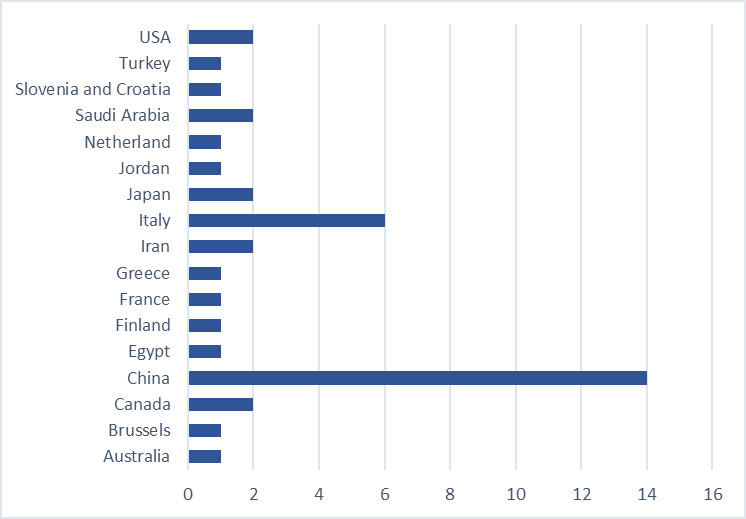


**Supplementary Figure S1: Publications on regional variations in predicting the success of m-TESE for NOA patients.**

This figure displays the number of publications from different countries focusing on predicting successful sperm retrieval in NOA patients undergoing m-TESE AI models. The figure highlights the research activity in various regions, indicating the volume of studies contributing to this area of prediction modelling.

Abbreviations: AI, Artificial Intelligence; m-TESE, Microdissection Testicular Sperm Extraction; NOA, Non-Obstructive Azoospermia.

**Supplementary Figure S2: PROBAST ROB assessment for studies predicting sperm retrieval in NOA patients undergoing m-TESE utilizing AI.**

Abbreviations: PROBAST, Prediction model Risk of Bias Assessment Tool; ROB, Risk of Bias; AI, Artificial Intelligence; m-TESE, Microdissection Testicular Sperm Extraction; NOA, Non-Obstructive Azoospermia.


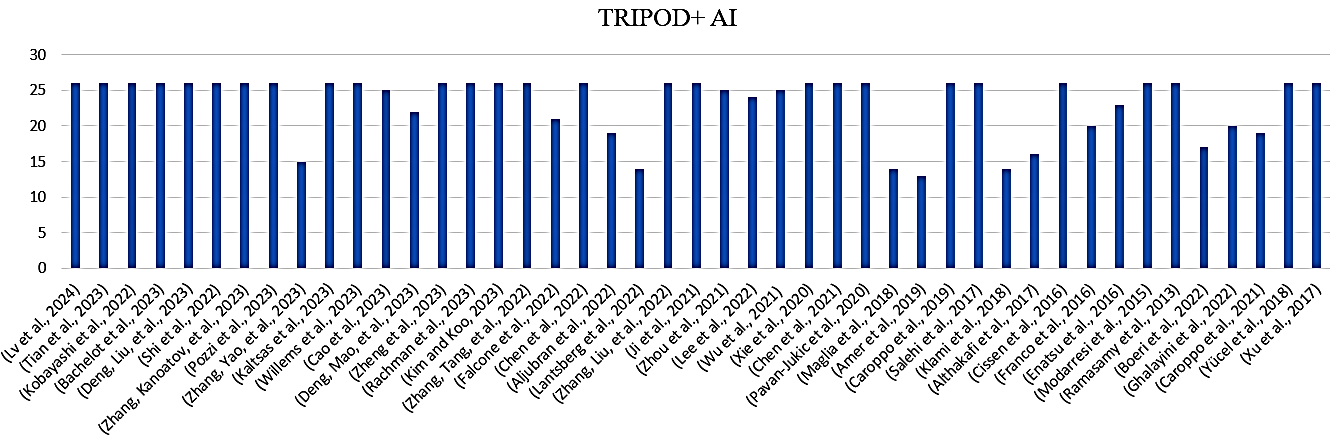


**Supplementary Figure S3:** **TRIPOD score across included studies.**

This figure illustrates the TRIPOD scores for all studies included in the review, providing a comprehensive assessment of the transparency and completeness of reporting in each study.

Abbreviations: TRIPOD, Transparent Reporting of a multivariable prediction model for Individual Prognosis or Diagnosis.
