# Supplementary material for: AI predictive models and advancements in microdissection testicular sperm extraction for non-obstructive azoospermia: a systematic scoping review
Source: Hum Reprod Open. 2024 Nov 21;2025(1):hoae070. doi: 10.1093/hropen/hoae070 (PMC11700607; doi:10.1093/hropen/hoae070)
Supplement: hoae070_Supplementary_Data [file hoae070_supplementary_data.zip › HRO-24-0251-R2-SuppTables1-2.docx]

**Supplementary Table S1: Search strategy scripts in PubMed and Scopus.**

| PubMed | Scopus |
| --- | --- |
| **((("Azoospermia"[Mesh]) AND "Artificial Intelligence"[Mesh]) AND "Sperm Retrieval"[Mesh]) OR ((("sperm retrieval?"[Title/Abstract] OR "testicular sperm retrieval"[Title/Abstract] OR "sperm retrieval, testicular"[Title/Abstract] OR "m?TESE"[Title/Abstract] OR "micro?dissection testicular sperm extraction"[Title/Abstract] OR "micro?TESE"[Title/Abstract] OR "testicular sperm extraction"[Title/Abstract] OR "sperm extraction"[Title/Abstract]) AND ("artificial intelligence"[Title/Abstract] OR "AI"[Title/Abstract] OR "Intelligen"*[Title/Abstract] OR "machine learning"[Title/Abstract] OR "ML"[Title/Abstract] OR "deep learning"[Title/Abstract] OR "neural network"[Title/Abstract] OR " artificial neural network"[Title/Abstract] OR "ANN"[Title/Abstract] OR "expert system"[Title/Abstract] OR "fuzzy logic"[Title/Abstract] OR "fuzzy"[Title/Abstract] OR "logistic regression"[Title/Abstract] OR "deep neural network"[Title/Abstract] OR DNN[Title/Abstract] OR "Data Mining"[Title/Abstract] OR "DM"[Title/Abstract] OR CNN[Title/Abstract] OR "convolution neural network"[Title/Abstract] OR predict*[Title/Abstract] OR predict*model*[Title/Abstract] OR predict*outcome*[Title/Abstract])) AND (non?obstructive azoospermia[Title/Abstract] OR NOA[Title/Abstract])** | TITLE-ABS-KEY ("sperm retrievals" OR "testicular sperm retrieval" OR “sperm retrieval, testicular" OR "m?tese"OR "microdissection testicular sperm extraction" OR "microTESE" OR "testicular sperm extraction" OR "sperm extraction" OR"TESE") AND TITLE-ABS-KEY ("artificial intelligence" OR "AI" OR intelligen* OR "machine learning" OR "ML" OR "deep learning" OR "neural network" OR "artificial neural network" OR "expert system" OR "fuzzy logic" OR "fuzzy" OR "logistic regression" OR "deep neural network" OR dnn OR "Data Mining" OR "DM" OR cnn OR "convolution neural network" OR predict* OR predict*model* OR predict*outcome*) AND TITLE-ABS-KEY ("non?obstructive azoospermia") OR "noa" ) ) |

**Supplementary Table S2: (Potential) Research gap(s) and limitation(s) of included studies.**

| **Author(s) and Year** | **Validation studies** | **Study design (accurate and reliable methods/ comparing another method/bias/ prospective)** | **Government policies/Standards** | **Randomized studies** | **Additional variables** | **Sample size** |
| --- | --- | --- | --- | --- | --- | --- |
| ***Machine Learning*** | | | | | | |
| Lv *et al.*, 2024 | YES | Not Mentioned | Not Mentioned | Not Mentioned |  | YES |
| Tian *et al.*, 2023 | Not Mentioned | Not Mentioned | Not Mentioned | Not Mentioned | YES | YES |
| Kobayashi *et al.*, 2022 | Not Mentioned | YES | Not Mentioned | Not Mentioned | Not Mentioned | Not Mentioned |
| Bachelot *et al.*, 2023 | Not Mentioned | YES | Not Mentioned | Not Mentioned | Not Mentioned | Not Mentioned |
| Deng, Liu, Z *et al.*, 2023 | Not Mentioned | Not Mentioned | Not Mentioned | Not Mentioned | YES | Not Mentioned |
| Shi *et al.*, 2022 | Not Mentioned | YES | Not Mentioned | Not Mentioned | Not Mentioned | Not Mentioned |
| Zhang, Kanoatov, *et al.*, 2023 | Not Mentioned | Not Mentioned | Not Mentioned | Not Mentioned | Not Mentioned | YES |
| Pozzi *et al.*, 2023 | Not Mentioned | Not Mentioned | Not Mentioned | Not Mentioned | YES | Not Mentioned |
| Zhang, Yao, *et al.*, 2023 | Not Mentioned | YES | Not Mentioned | Not Mentioned | Not Mentioned | Not Mentioned |
| Kaltsas *et al.*, 2023 | Not Mentioned | YES | Not Mentioned | Not Mentioned | YES | Not Mentioned |
| Willems *et al.*, 2023 | Not Mentioned | YES | Not Mentioned | Not Mentioned | Not Mentioned | Not Mentioned |
| Cao *et al.*, 2023 | Not Mentioned | Not Mentioned | Not Mentioned | Not Mentioned | YES | Not Mentioned |
| Deng, Mao, *et al.*, 2023 | Not Mentioned | YES | Not Mentioned | Not Mentioned | Not Mentioned | YES |
| Zheng *et al.*, 2023 | YES | YES | Not Mentioned | Not Mentioned | Not Mentioned | YES |
| Rachman *et al.*, 2023 | YES | YES | Not Mentioned | Not Mentioned | Not Mentioned | YES |
| Kim and Koo, 2023 | YES | YES | Not Mentioned | Not Mentioned | Not Mentioned | YES |
| Zhang, Tang, *et al.*, 2022 | Not Mentioned | YES | Not Mentioned | Not Mentioned | Not Mentioned | Not Mentioned |
| Falcone *et al.*, 2022 | Not Mentioned | YES | Not Mentioned | YES | Not Mentioned | Not Mentioned |
| Chen *et al.*, 2022 | Not Mentioned | YES | Not Mentioned | Not Mentioned | YES | YES |
| Aljubran *et al.*, 2022 | Not Mentioned | Not Mentioned | Not Mentioned | Not Mentioned | YES | YES |
| Lantsberg *et al.*, 2022 | Not Mentioned | YES | Not Mentioned | Not Mentioned | YES | Not Mentioned |
| Zhang, Liu, *et al.*, 2022 | Not Mentioned | Not Mentioned | Not Mentioned | Not Mentioned | YES | Not Mentioned |
| Ji *et al.*, 2021 | YES | Not Mentioned | Not Mentioned | Not Mentioned | Not Mentioned | YES |
| Zhou *et al.*, 2021 | YES | Not Mentioned | Not Mentioned | Not Mentioned | Not Mentioned | YES |
| Xie *et al.*, 2020 | Not Mentioned | YES | YES | Not Mentioned | Not Mentioned | YES |
| Chen *et al.*, 2021 | YES | Not Mentioned | Not Mentioned | Not Mentioned | Not Mentioned | Not Mentioned |
| Pavan-Jukic *et al.*, 2020 | Not Mentioned | YES | Not Mentioned | Not Mentioned | YES | Not Mentioned |
| Maglia *et al.*, 2018 | Not Mentioned | Not Mentioned | Not Mentioned | YES | YES | Not Mentioned |
| Amer *et al.*, 2019 | YES | Not Mentioned | Not Mentioned | Not Mentioned | Not Mentioned | Not Mentioned |
| Caroppo *et al.*, 2019 | Not Mentioned | YES | Not Mentioned | Not Mentioned | Not Mentioned | YES |
| Salehi *et al.*, 2017 | Not Mentioned | YES | Not Mentioned | Not Mentioned | Not Mentioned | Not Mentioned |
| Klami *et al.*, 2018 | Not Mentioned | YES | Not Mentioned | Not Mentioned | Not Mentioned | Not Mentioned |
| Althakafi *et al.*, 2017 | Not Mentioned | Not Mentioned | Not Mentioned | Not Mentioned | YES | Not Mentioned |
| Cissen *et al.*, 2016 | Not Mentioned | Not Mentioned | YES | YES | YES | Not Mentioned |
| Franco *et al.*, 2016 | Not Mentioned | Not Mentioned | Not Mentioned | Not Mentioned | Not Mentioned | YES |
| Enatsu *et al.*, 2016 | Not Mentioned | YES | Not Mentioned | Not Mentioned | Not Mentioned | YES |
| Modarresi *et al.*, 2015 | Not Mentioned | Not Mentioned | Not Mentioned | Not Mentioned | YES | Not Mentioned |
| Ramasamy *et al.*, 2013 | Not Mentioned | YES | Not Mentioned | Not Mentioned | YES | Not Mentioned |
| **Salvage** | | | | | | |
| Boeri *et al.*, 2022 | YES | YES | Not Mentioned | Not Mentioned | Not Mentioned | YES |
| Ghalayini *et al.*, 2022 | Not Mentioned | YES | Not Mentioned | Not Mentioned | Not Mentioned | Not Mentioned |
| Caroppo *et al.*, 2021 | Not Mentioned | YES | Not Mentioned | Not Mentioned | Not Mentioned | Not Mentioned |
| Yücel *et al.*, 2018 | Not Mentioned | YES | Not Mentioned D | Not Mentioned | Not Mentioned | YES |
| Xu *et al.*, 2017 | Not Mentioned | YES | Not Mentioned | Not Mentioned | Not Mentioned | YES |
| **Deep Learning** | | | | | | |
| Lee *et al.*, 2022 | Not Mentioned | YES | Not Mentioned | Not Mentioned | Not Mentioned | Not Mentioned |
| Wu *et al.*, 2021 | Not Mentioned | YES | Not Mentioned | Not Mentioned | Not Mentioned | Not Mentioned |
